# Corruption in Anglophone West Africa health systems: a systematic review of its different variants and the factors that sustain them

**DOI:** 10.1093/heapol/czz070

**Published:** 2019-08-04

**Authors:** Obinna Onwujekwe, Prince Agwu, Charles Orjiakor, Martin McKee, Eleanor Hutchinson, Chinyere Mbachu, Aloysius Odii, Pamela Ogbozor, Uche Obi, Hyacinth Ichoku, Dina Balabanova

**Affiliations:** 1 Health Policy Research Group, Department of Pharmacology and Therapeutics, College of Medicine, University of Nigeria, Enugu-Campus, Enugu, Nigeria; 2 Department of Health Administration and Management, University of Nigeria, Enugu-Campus, Enugu, Nigeria; 3 Department of Social Work, University of Nigeria, Nsukka, Nigeria; 4 Department of Psychology, University of Nigeria, Nsukka, Nigeria; 5 Department of Global Health and Development, London School of Hygiene and Tropical Medicine, 15-17 Tavistock Place, London WC1H 9SH, UK; 6 Department of Community Medicine, College of Medicine, University of Nigeria, Enugu-Campus, Enugu, Nigeria; 7 Department of Sociology, University of Nigeria, Nsukka, Nigeria; 8 Department of Economics, University of Nigeria, Nsukka, Nigeria

**Keywords:** Health sector, health sector corruption, African health systems, Anglophone West Africa, universal health coverage

## Abstract

West African countries are ranked especially low in global corruption perception indexes. The health sector is often singled out for particular concern given the role of corruption in hampering access to, and utilization of health services, representing a major barrier to progress to universal health coverage and to achieving the health-related Sustainable Development Goals. The first step in tackling corruption systematically is to understand its scale and nature. We present a systematic review of literature that explores corruption involving front-line healthcare providers, their managers and other stakeholders in health sectors in the five Anglophone West African (AWA) countries: Gambia, Ghana, Liberia, Nigeria and Sierra Leone, identifying motivators and drivers of corrupt practices and interventions that have been adopted or proposed. Boolean operators were adopted to optimize search outputs and identify relevant studies. Both grey and published literature were identified from Research Gate, Yahoo, Google Scholar, Google and PubMed, and reviewed and synthesized around key domains, with 61 publications meeting our inclusion criteria. The top five most prevalent/frequently reported corrupt practices were (1) absenteeism; (2) diversion of patients to private facilities; (3) inappropriate procurement; (4) informal payments; and (5) theft of drugs and supplies. Incentives for corrupt practices and other manifestations of corruption in the AWA health sector were also highlighted, while poor working conditions and low wages fuel malpractice. Primary research on anti-corruption strategies in health sectors in AWA remains scarce, with recommendations to curb corrupt practices often drawn from personal views and experience rather that of rigorous studies. We argue that a nuanced understanding of all types of corruption and their impacts is an important precondition to designing viable contextually appropriate anti-corruption strategies. It is a particular challenge to identify and tackle corruption in settings where formal rules are fluid or insufficiently enforced.

**Key Messages**

- Health sector corruption in Anglophone West Africa is judged prevalent, contributing to the region’s appalling ratings in global corruption perception indexes.
- Recorded top corruption concerns marring efficiency of health systems in Anglophone West Africa are absenteeism, diversion of patients to private facilities, inappropriate procurement, informal payments and theft of medical supplies.
- Frequently occurring drivers of these corruption concerns include weak value systems, normalization of inappropriate practices, information asymmetry and poor staff welfare/working conditions.
- Interventions to curb recorded corruption concerns were largely recommended, which opens up the path for future studies to empirically explore interventions that would be feasible.

## Introduction

Corruption is endemic in many low- and middle-income countries (LMICs), including those in Anglophone West African (AWA), affecting all sectors but especially health (World Bank, 2015; World Bank, n.d.). It undermines health service delivery, entrenching inequalities in access and encouraging inappropriate care, sometimes with lethal consequences (Dovlo, 2012; World Bank, 2015; Akokuwebe and Adekanbi, 2017). It is a major barrier to achieving the health-related Sustainable Development Goals, including universal health coverage. Yet despite its undoubted importance, solutions have been elusive, in part because it is complex and takes place away from public gaze (Human Rights Watch, 2007; Holeman *et al.*, 2016; Hadi, n.d.). Consequently, a first step in tackling corruption is to bring it into the light, revealing its myriad forms and the factors that have allowed it to persist in the face of attempts to combat it. Doing so will help policymakers to develop and evaluate evidence-based responses.

Transparency International (TI) defines corruption as the abuse of entrusted power for private gain (Transparency International, 2019). de Sardan (2013) further posits that corruption is manifest in the divergence between actual behaviours of employees and official norms governing them. In many cases, health system actors diverge from official regulations and norms, which may be constraining and poorly designed, in part as a coping mechanism in poorly resourced health systems. It can be argued that rule-breaking, non-compliant practices that have neutral or even beneficial impacts on patient care need to be delineated from harmful informal/illicit practices (corruption) (Khan *et al.*, 2016). Thus, Vian (2008) describes health sector corruption as rule-breaking practices abetted by front-line health workers, facility managers and governmental authorities (see has health system agents) that put health service users at high risk of not receiving accessible or appropriate care. Vian (2008) not only acknowledges that corrupt practices may be triggered by health service users, but also describes how corruption occurs in circumstances when particular practices have evolved to become system-wide norms with popular acceptance. This review thus explores the types and drivers of corruption that not only breach rules and norms but also (potentially or actually) harm access to care and health outcomes.

Corruption in health sectors in AWA has been linked to many adverse outcomes, including reduced efficiency of health systems and increased mortality (Vian and Norberg, 2008; Adegboyega and Abdulkareem, 2012; Onotai and Nwankwo, 2012; World Bank, 2015; Gaitonde *et al.*, 2016; Mooketsane and Phirinyane, 2017). The scale of the problem in AWA is apparent from corruption perception indices published annually by TI. Countries in this sub-region are consistently rated poorly. The 2018 Corruption Perceptions Index (CPI) ranked Ghana in 81st position out of 180 countries, with a score of 51% (where 100% is full transparency). Liberia ranks 122nd, at 32%, followed by Gambia and Sierra Leone, tied in 130th position, with 28%, while Nigeria lagged behind in 148th position, with a score of 18%. In TI’s ratings, the health sector is consistently reported as one of the most corrupt sectors, in part reflecting the private nature of many of the interactions between users and providers and the asymmetry of information involved.

While there is widespread, often anecdotal, recognition that corruption in health sectors is common, there is much less information on its nature and dynamics in individual countries. In this article, we address this problem by reviewing systematically what is known about corruption within health systems in AWA. The importance of doing so is not in doubt, given the imperative of getting maximum value from the limited resources provided for health in this sub-region and the increasing demands for transparency imposed by the development agencies that are so important in supporting health services. Yet these efforts are constrained by the lack of evidence on the types of corruption in the health sector, the forms that they take and the drivers that sustain corrupt practices in the health sector. Khan argued that a better understanding of the nuanced nature of corruption can also facilitate the shift from historical, and largely unsuccessful vertical, normative government-driven anti-corruption measures that de-emphasize the involvement of those on the front-line, such as health workers and facility managers (Khan, 2017). Instances of vertical enforcement measures include policies to regulate drug distribution and sales, fire or penalize health workers, adapt salary structure, financial audits by government-mandated anti-corruption bodies, etc. Horizontal actions include, e.g. monitoring of attendance registers by facility heads, sensitizing health consumers to their rights, community monitoring by locally established committees, monitoring internally generated revenue, etc. Consequently, we seek to understand how health workers circumvent anti-corruption strategies, what can incentivize them to follow the rules (horizontal approaches), and thereby to identify plausible responses that can be implemented locally.

Our findings will inform health researchers, policymakers and donors as they seek to understand how corruption affects health system performance and help identify interventions that can combat corruption in the health sector. This is an important given paucity of evidence on how best to eliminate the factors that encourage and sustain corruption.

Proposed anti-corruption measures included stringent sanctions on doctors who accept kickbacks and bribes from pharmaceutical industries, mechanisms to increase visibility of informal payments and strict enforcement of existing rules in health facilities (Dabo *et al.*, 2014; Gaitonde *et al.*, 2016; Mackey *et al.*, 2016). However, very little is known about whether and in what circumstances these measures work. Thus, there is clear need to examine what evidence does exist to inform context-specific anti-corruption strategies and policies.

This article reviews and synthesizes knowledge on the nature and scope of corruption in the health sector amongst health workers who have been reported in published literature on AWA countries. It identifies the types of corruption that exist in their health sectors, examines the incentives that give rise to corruption, inappropriate and ineffective care by front-line healthcare providers (those who interact with patients) and their managers and suggests measures that might possibly reduce or eliminate these incentives.

## Methods

We conducted a systematic review of published material on corruption in health systems in the AWA countries, comprising Gambia, Ghana, Liberia, Nigeria and Sierra Leone. All the AWA countries are classified by the World Bank as LMICs. The search strategy employed was as inclusive as possible, recognizing that an over-restrictive approach could compromise our quest to achieve a full understanding of the nature of this phenomenon. It used a series of key search terms developed following extensive consultation within the research team and applying appropriate Boolean operators.

The searches were conducted following adaptation to the features of the databases, in PubMed, Researchgate, Hinari and Google Scholar. Studies were included initially if they were: (1) published between 2007 and 2017; (2) focused on corruption within AWA countries; and (3) written in English or with an available English translation. Each publication was examined independently by three members of the team to determine duplication and relevance before data extraction. Then, we categorized the different types of corruption; identified causes or processes involved; factors that facilitate different forms of corruption; the effects of corruption; and interventions that have been suggested or implemented. Given the wide diversity of literature reviewed, some of which were expected to be merely descriptive, it was inappropriate to undertake a formal risk of bias assessment.

### Conceptual frameworks

Three existing conceptualizations of corruption guided our review and synthesis (see an overview in Table 1). The first, drawing on work by Vian (2008), focuses on understanding how opportunities, pressures on and rationalizations by key actors influence corruption. Based on this, we identified areas of the health sector prone to corruption, including provision of services by medical personnel, human resources management, drug selection and use, procurement of drugs and medical equipment, distribution and storage of drugs, regulatory systems, budgeting and pricing. We also drew on another actor-oriented conceptualization of health sector corruption, by Gaitonde *et al.* (2016), emphasizing actions and inactions of stakeholders within the health system as key to sustaining corruption (Table 2).

**Table 1:** Conceptualizations of corruption informing the study

S/no.	Author(s)	Basic assumption	Explanation
1	Vian (2008) and Gaitonde *et al.* (2016)	Corruption in the health sector is caused and sustained by ‘key stakeholders who are either opportunists, pressured, or good at rationalizing’ supposed corrupt practices as norms. [*vertical approaches/regulation solutions*]	The consequence of actions and inactions of stakeholders in the health sector has severely corrupted the system, particularly in the area of selection, procurement and distribution of drugs, health financing and human capital management.
2	Vian and Norberg (2008)	‘Relationships among stakeholders’ (based on social norms, pragmatic objectives or other reciprocal relationships) in the health sector form a strong conduit for corruption. [*vertical approaches/ regulation solutions*]	Corruption happens when government agents engage inappropriate practices because the health system is poorly governed; clients are deprived of healthcare which naturally should be their rights, and so are pressured to pay bribes or take to other ill processes in seeking health services. Whereas, health workers/managers are forced to in same failing system, preferentially cater for those they share social ties with.
3	de Sardan (2013) and Gaal and McKee (2004)	Behaviours of stakeholders in the health sector that deviate from ethics and principles ‘are mostly informal’ and often at the junction of what is considered ‘the usual practice’ and corruption. Gaal and McKee further argue that consumers and providers, instead of seeking an official recourse to get a service (‘voice’), or seeking care outside the public sector (‘exit’), resort to informal means to achieving their objectives (giving an informal payment or gift) within the limits of the existing system (informal exit or ‘inxit’). [*horizontal approaches/collective action solutions*]	Informal behaviours of health sector stakeholders cause corruption to thrive, and affect health service users and workers who are less powerful. The less powerful groups can challenge these informal behaviours if given a voice. If not, they can disengage from the process. Thus, the scaling up the voice of less powerful groups affected by such informal behaviours can be a basis for anti- corruption activities.

**Table 2:** Types, interactions and mechanisms of corruption in health system

Types of behaviour	Types of interactions			
	With government regulators	With payers		With patients
	Between government regulators and suppliers, payers or providers	Between payers and suppliers	Between payers and providers	Between suppliers, providers and patients
Theft (taking resources without permission or right)	Collusion in embezzlement (fraudulent appropriation of resources) by government regulators	Embezzlement by suppliers	Embezzlement by managers in provider organizations	Sale of drugs or supplies that were supposed to be free by health workers
		Not delivering on a contract by suppliers	Not delivering on a contract by provider organizations	
			Pilfering of supplies by health workers	
			Private use of public facilities and equipment by health workers	
Bribes (giving or taking money or something else of value to influence a decision for private gain)	Bribes to obtain regulatory decisions benefiting suppliers, payers or providers (including state capture)	Bribes or kickbacks to obtain contracts benefiting suppliers	Bribes or kickbacks to obtain contracts benefiting providers	Informal payments by patients to doctors to obtain access or quality
	Bribes to obtain accreditation, certification, approval (e.g. drug registration), or inspection results		Fee-splitting by specialists to referring health workers to induce referrals	
	Policy decisions to further public officials’ or politicians’ careers			
Misinformation (falsifying information for private gain)	False reporting by suppliers, payers or providers to government regulators	Falsifying information to obtain contracts benefiting suppliers	False insurance claims	Falsification of credentials by health workers
			Prescription fraud (bogus or forged prescriptions to bill payers)	Supplier-induced or supplier-reduced demand
			Absenteeism (spending less time than contracted to deliver care)	Misleading promotion of drugs/products to patients
			Misleading drug promotion to prescribers, including pseudo-trials used to market drugs	

Source: Gaitonde *et al.* (2016).

The second conceptualization, which underpins the Vian and Norberg (2008), argues that corruption thrives in health systems because of the nature of relationships among stakeholders in the system. These can involve exchanges, practical gains and social norms. Vian (2008) identified three groups of agents who can encourage corruption in the health sector: government agents who adopt corrupt practices in response to failings in the health system; pressured clients who would do anything to get quicker and better quality health services; and health workers/managers who struggle with competing pressures from their families and others. Vian also notes how social norms can sustain corruption in the health sector, with inappropriate practices being tolerated and eventually normalized.

Thirdly, de Sardan (2013) theorizes corruption as informal behaviours that contradict official norms, with consequences mostly for the less powerful or disadvantaged groups. He believes that informal activities amount to rule breaking, and so portend risks to both health workers and service consumers who are less powerful. This idea is also captured in Gaal and McKee’s (2004) interpretation of Hirschman’s theory of consumer behaviour where health service users engage in informal and illicit behaviours (where required) in order to achieve their objectives within a system that is under resourced and where legitimate claims are not respected (informal exit or ‘inxit’). These conceptualizations suggest that effective action would involve giving less powerful groups voice to transform the current system.

The three conceptualizations differ in terms of their emphasis on vertical approaches (better regulations, sanctions and structured incentives) vs. horizontal approaches (involving flexible incentives and collective action solutions). A summary is presented in Table 1.

### Findings

**Figure 1:**
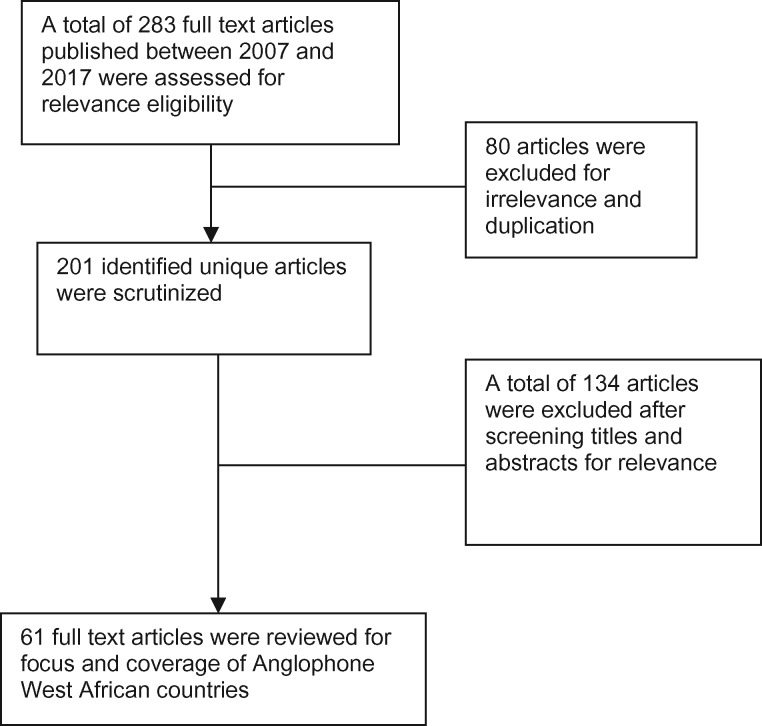
Flow chart showing detailed article extraction and evaluation method.

In total, 283 published, unpublished and grey literature reports were retrieved initially from the primary searches. After initial screening, 61 were found to match the inclusion criteria and were retained and reviewed. The flow chart describing this process is shown in Figure 1. The largest single category comprised academic journal articles (*n* = 29). Other sources included technical reports from civil society organizations, students’ projects, news articles and book chapters.

None of the publications included were on Gambia or Liberia. Most were published from 2010 onwards. The studies employed diverse methodological approaches, using a range of quantitative and qualitative methods.

We first identify the causes of corruption in the health sector, to enable an understanding of motivations for different types of corrupt practices in the health sector and the effects each could have on health systems in the region. We then discuss ways to curb these practices. The prevalence of different corrupt practices in the health sector, agencies that reinforce them, dimensions they take and drivers/causes are synthesized in Table 3, alongside corresponding interventions.

**Table 3:** Analysis of corruption in the health sector (types, agencies, dimensions, causes and consequences) and potential interventions

Type or form of corruption	Agency	Dimensions	Causes	Consequences	Intervention
Informal payments and bribery	Clients to health workers	Service delivery	Desire to jump queue Quest for preferential care Ignorance about rights	Differential quality of care Denial of care Normalization of corruption	Public display of price list Client sensitization of rights
	Clients and health managers	Payment mechanism	Direct cash payment	Lack of trust (clients to HWs) Financial risk	ICT-based monitoring of payments for services
	Health workers and government	Human resource management	Poor and irregular salaries		Incentives and better remuneration
Absenteeism	Health workers and health managers	Service delivery HR management	Dual practice Lack of enforcement of regulations Poor work environment Geographic access Gender roles Political ‘protection’	Poor utilization of public health facilities – (poor health outcomes) Reduced satisfaction with care Prolonged waiting times	Community monitoring of health workers’ compliance with work ethics Rewards (performance bonuses) and sanctions
Theft and diversion of drugs and supplies from public facilities	Health workers	Pharmaceuticals Service delivery	Absence (or weak enforcement) of consumer protection laws	Shortage of medical supplies Wastage of public resources	Regular monitoring of stores, store records and procedures Partnership with security and other agencies
Unauthorized procurement processes/procurement irregularities	Government, health managers, health workers, pharmaceutical companies/reps	Pharmaceuticals Budget and Pricing Regulation	Absence (or weak enforcement) of consumer protection laws	High cost of providing health services Inadequate and poor quality of health equipment	Automated procurement processes Transparent pricing lists for services and consumables
Diversion of patients from public to private hospitals and vice versa	Health workers and clients	Service delivery	Dual practice Poor pay Information asymmetry Political protection of doctors	Wastage of public resources Exorbitant fees for patients Deprivation of healthcare for other patients	Enact and enforce anti-corruption laws in health Improved pay and work conditions for health workers
Employment and staff development irregularities	Government, health managers and health workers	HR management		Poor staff development	Enact and enforce anti-corruption laws in health
Mismanagement and misappropriation of resources (incl. money)	Government and health managers	Budget and pricing	Poor documentation	High cost of providing health services Wastage of public resources	Transparent pricing lists for consumables and services
Fraud and forgery (related to audit processes)	Government and health managers	Regulation	Inadequate monitoring		Enact and enforce anti-corruption laws in health

### Drivers of corruption

Several characteristics of health systems in the region facilitate corrupt practices. One is the role of direct payments, in cash or in kind for in-health-related transactions (Onwujekwe *et al.*, 2010; Kamorudeen and Bidemi, 2012; Onotai and Nwankwo, 2012; Onah and Govender, 2014; Kankeu and Ventelou, 2016; Saka *et al.*, 2016; Akokuwebe and Adekanbi, 2017; Hoffman and Patel, 2017). In-kind payments are more common in rural areas, where poverty persists and patients or their relatives may do menial jobs for health workers (Onah and Govender, 2014). In addition, the exclusion of many people from insurance schemes increases the frequency of out-of-pocket payments, which can easily be linked to demands for extra informal contributions (Aregbesola, 2016).

A second is the lack of systems of patient rights (Hussmann, 2010) but, even when they exist, patients may be unaware of their rights and of the legal procedures for redress (Vian, 2008). Thus, there are few formal channels for patients to challenge health workers (Onwujekwe *et al.*, 2010; Ojiaku, 2014; Hoffman and Patel, 2017).

A third is the widespread shortage of many of the inputs to health systems. For instance, when medical consumables are in short supply, health workers felt justified in overcharging patients who are in dire need of them (Akokuwebe and Adekanbi, 2017). Physicians are often absent from public health facilities, reflecting low wages and irregular payment of salaries (Onwujekwe *et al.*, 2010; Ojiaku, 2014; Hoffman and Patel, 2017). Many are involved in dual practice, seen as contributing to widespread absenteeism (Kamorudeen and Bidemi, 2012; Chimezie, 2015). Physicians often rationalize dual practice as a means to improve their low earnings and public hospitals may tolerate this practice because it avoids pressure to increase salaries (Vian, 2008; Aregbesola, 2016). Even when health workers employed in the public sector are banned from engaging in private practice during work hours, some continue to do so (Aregbesola, 2016). Kamorudeen and Bidemi (2012) identified a widespread view among patients that a relationship with health workers secured through bribery was necessary, in these circumstances, to obtain quick, acceptable quality healthcare. Finally, corrupt behaviours are facilitated by poor documentation and weak systems of oversight and governance (Vian, 2008; Stakeholder Democracy Network, 2013).

Although we have focused our review on horizontal processes and relationships that are most amenable to local action, it is important to note that action is often constrained by the wider political aspects of corruption. Corrupt practices were frequently attributed to the nature of the relationships between officials and politicians. Some government practices encourage corruption, including exploitation of healthcare facilities for political purposes (Akpomuvie, 2010), as when they are situated in locations that offer political benefits to those making the decisions rather than meeting the health needs of the population. Inadequate health-budget-led supply failures and low pay also creates conditions in which various forms of corruption can flourish (Agbenorku, 2012; Vian and Norberg, 2008). These various forms are further discussed below.

### Types/forms of corruption in the health sector

Five main types of corruption were identified. They are: (1) bribery/informal payments to health providers; (2) absenteeism and late arrival to work; (3) theft and diversion of drugs and other supplies/equipment from the public to private facilities; (4) inappropriate procurement of medical consumables and equipment; (5) diversion of patients from public facilities to health workers’ private facilities and vice versa. These often co-existed. To illustrate the different types of corruption, Table 4 categorizes them by country, whereas Table 5 summarizes the processes involved in each type of corruption and interventions that have been proposed to combat them. We now discuss each in turn.

**Table 4:** Categorization of the main types and interventions to curb health sector corruption based on West African Anglophone countries

S/no.	Country	No of papers	Main types/causes of corruption	Authors that explored different types of corruption	Main interventions of corruption	Authors that elaborated on different interventions
1	Nigeria	50	Absenteeism (a) Wages of health workers are often low leading to them seeking other means to make ends meet. Oftentimes, they are absent from work as a result of this.	Akwataghibe *et al.* (2013); Mackey *et al.* (2016); Maduke (2013); UNDP (2011); Maduke (2013); Azuh (2012); Stakeholder Democracy Network (2013); Chimezie (2015); Ojiaku (2014); Tormusa and Idom (2016); Vian and Norberg (2008); Aregbesola (2016); and Onwujekwe *et al.* (2010)	(a) Proper motivational incentives should be provided for health workers and their salary structure should be reviewed as well.	Ojiaku (2014); Maduke (2013); Onwujekwe *et al.* (2010); Agbenorku (2012); Stakeholder Democracy Network (2013); Vian and Norberg (2008); Saka *et al.* (2016); and Vian and Norberg (2008)
			(b) Poor governance structures	Mooketsane and Phirinyane (2017); World Bank (2015); Akpomuvie (2010); Vian and Norberg, 2008; Onotai and Nwankwo (2012); Saka *et al.* (2016); and Vian (2008)	(b) Public office holders in West Africa must be accountable and transparent in their discharge of duty. More also, adequate measures in form of checks and balances coupled with effective monitoring and evaluation of health resources and outcomes in should be put in place to halt corrupt practices in the health sector.	Mooketsane and Phirinyane (2017); Gaitonde *et al.* (2010); Azuh (2012); Onotai and Nwankwo (2012); UNDP (2011); Adegboyega and Abdulkareem (2012); World Bank (2016); Holeman *et al.* (2016); Onuigbo and Eme (2015); Kamorudeen and Bidemi, (2012); and Mackey *et al.* (2016)
			(c) Weak accountability systems and the normalization of corrupt practices	Amnesty International (2011); Adegboyega and Abdulkareem (2012); Stakeholder Democracy Network (2013); Hoffman and Patel (2017); Vian (2008); Tormusa and Idom (2016); and Maduke (2013)	(c) Relevant supervisory agencies should be put in place to ensure administrative compliance to stated rules in the organization. Also, anti-corruption initiatives that includes withdrawal from service, whistle blowing mechanism, sanctions, etc. should be adopted	Tormusa and Idom (2016); Kamorudeen and Bidemi, (2012); Vian (2008); UNDP (2011); Bloom et al(2012); Hoffman and Patel (2017); and Akokuwebe and Adekanbi (2017);
			(d) Out of stock: frequent cases of insufficient items like drugs were reported even though mechanisms were put in place to prevent it, e.g. drug revolving fund	Azuh (2012) and Saka *et al.* (2016)	(d) Awareness creation through seminars and symposiums on the dangers of corruption in the health sector and on their image as health workers.	Akokuwebe and Adekanbi (2017) and Hoffman and Patel (2017)
			(e) Poor working conditions	Stakeholder Democracy Network (2013)	(e) making sure that adequate equipment for effective service are made available	Stakeholder Democracy Network (2013) and Akokuwebe and Adekanbi (2017)
			(f) Bribes/informal payments: belief that one cannot access quality healthcare unless one makes informal payments to healthcare providers or one is known by them	Akokuwebe and Adekanbi (2017); Hoffman and Patel (2017); Kamorudeen and Bidemi, (2012); Saka *et al.* (2016); Turay (2016); Adegboyega and Abdulkareem (2012); Chimezie (2015); Azuh (2012); Hoffman and Patel (2017); Gaitonde *et al.* (2010); and Kankeu and Ventelou (2016)	(f) Drugs and services that are free should be made obvious and official pricing policies should be known by patients and also, they should be made aware of the possibility of health providers overcharging them and as such proper platform for reporting should be provided so they can leverage on it to report cases of overcharging.	Maduke (2013); Akokuwebe and Adekanbi (2017); Hoffman and Patel (2017); and Onwujekwe *et al.* (2010)
			(g) Motivational incentives such as allowance, training, etc. that are not fully implemented	Ojiaku (2014)	(g) empowering independent agencies to investigate and enforce cases of corruption in the health sector Provision of new and practicable incentive mechanisms	Vian (2008); Gaitonde *et al.* (2010); Akinbajo (2012); Delanyo (2012); Maduke (2013); and Amnesty International (2011)
			(h) The various actors that interact in the process of health delivery is a catalyst for corruption	Vian (2008)	(h) Community based health insurance schemes can help eliminate cases of corruption in the health sector.	Onotai and Nwankwo (2012) and Agbenorku (2012)
			(i) Lack of emphasis on quality service delivery	World Bank (2015); Vian and Norberg, 2008; and Stakeholder Democracy Network (2013)	(i) Community monitoring is an effective strategy that ensures that there is accountability in the work place. Moreover, it helps reduce the problem of medicine stock, absenteeism, informal payments, and other forms of corrupt practice in the health sector.	Vian and Norberg (2008); Alenoghena *et al.* (2014); UNDP (2011); and Bloom *et al.* (2011)
			(j) Information asymmetry	Kamorudeen and Bidemi (2012); WHO (2016); Vian and Norberg, 2008; WHO (2016); and Matsheza *et al.* (2011)	(j) Open contracting helps to bridge the problem of information asymmetry and therefore should be used and also, it helps to ensure that needs assessments are published and that contracts are completed in timely order.	WHO (2016) and Mackey *et al.* (2016)
2	Ghana	9	(a) People pay bribes for jumping the queue, receiving better or more care, obtaining drugs or just simply for any care at all.	Agbenorku (2012) and The Association of Chartered Certified Accountants (2013)	(a) Motivation and payment of good salaries to health workers and incentives to health workers.	Agbenorku (2012)
			(b) There is selling of public positions and payment of bribes to get promotion. Newly hired and promoted must find the resources to ensure their continued employment and advancement.		(b) The use of education as a necessary tool to sensitize the public and help fight this ‘norm’.	Dizon-Ross *et al.* (2017); UNDP (2011); and Agbenorku (2012)
			(c) Inadequate salaries for health workers	Agbenorku (2012) and Maduke (2013);	(c) The centralized hiring, promotion and deployment of public health workers in all countries effectively neutralizes the role of local supervision.	
			(d) Unavailability of sufficient tools to work with	Agbenorku (2012)	(d) Provision of adequate equipment for effective service delivery	Agbenorku (2012) and The Association of Chartered Certified Accountants (2013)
			(e) Illegal money payment has become part of the requirements before one can access any service needed.	Dizon-Ross *et al.* (2017) and Agbenorku (2012)	(e) Conduct regular health provider audits by the central government which has been proven to encourage more responsible public services and regular checks on staff	Vian (2008); WHO (2016); UNDP (2011); and The Association of Chartered Certified Accountants (2013)
			(f) Imbalances of policy decision-making power related to strong and dominant political actors combined with weak civil society engagement, accountability systems and technical analyst power and position.		(f) The government should promote good governance and people are held accountable for their offences. Strict measures to be explored including total withdrawal from service	Agbenorku (2012); Mooketsane and Phirinyane (2017); Vian (2008); Holeman *et al.* (2016); and Agyepong (2008)
			(g) Information asymmetry	WHO (2016)	(g) Public sensitization for healthcare receivers	WHO (2016); Dizon-Ross *et al.* (2017); and Agbenorku (2012)
			(h) Unmonitored National Health Information Scheme		(h) Application of Information Communication Technology to monitoring NHIS	The Association of Chartered Certified Accountants (2013)
			(i) Poor working conditions	WHO (2016) and Agbenorku (2012)		
3	Sierra Leone	9	(a) Reluctance by health sector managers and administrators to instil values of integrity, transparency and accountability in the sector.	Pietersen and Lodge (2015) and Turay (2016)	(a) Institute anti-corruption measures consistent with the country’s National Anti-Corruption Strategy 2014–18. The strategy provides for fighting corruption by MDAs taking ownership of the fight within their respective institutions, which requires the setting up of integrity management committees within the health sector.	National Anti-Corruption Strategy (Sierra Leone) 2014–2018 and Turay (2016)
			(b) Underpaid medical staff, qualified staff are made to work for years as volunteers and when salaries are paid, its shockingly low.	Mitchell (2017)	(b) Government instructs placement of posters at the state run hospital in the centre of town which proclaim ‘pay no bribe’, urging people to report any cases of bribery they may encounter. This new initiative allows anonymous reporting is a great innovation, organized by Sierra Leone’s Anti-Corruption Commission, and funded by the UK Department for International Development, allows people to call a toll-free number to report cases of corruption across the education, electricity, health, police, water and sanitation sectors. This innovation has gone some way to putting some of the power back into the hands of the people using these services.	Mitchell (2017)
			(c) A lack of accountability	Turay (2016)	(c) Better pay for nursing staff and other health workers	Mitchell (2017)
			(d) Information asymmetry	National Anti-Corruption Strategy (Sierra Leone) 2014–2018 and Turay (2016)	(d) Tighter regulation of hospitals and scrutiny of healthcare budgets	Mitchell (2017) and Turay (2016)
			(e) Understaffing		(e) Employment of more staff	
			(f) Poor working conditions.	‘Pay No Bribe’ (PNB) programme; Turay (2016)		

**Table 5:** Summary of area of processes, types and resultant effects of corruption in the study

	Area of process	Type	Studies from our review (authors and years)	Effective strategies to combat corruption in the health sector
1	Construction and rehabilitation of health Facilities	Bribes, kickbacks and political considerations influencing the contracting process; contractors fail to perform and are not held accountable	Mooketsane and Phirinyane (2017); Akpomuvie (2010); Vian and Norberg (2008); and Onotai and Nwankwo (2012)	High cost, low-quality facilities and construction work; location of facilities that does not correspond to need, resulting in inequities in access; biased distribution of infrastructure favouring urban- and elite-focused services.
2	Purchase of equipment and supplies, including drugs	Bribes, kickbacks and political considerations influence specifications and winners of bids; collusion or bid rigging during procurement; lack of incentives to choose low cost and high-quality suppliers; unethical drug promotion Suppliers fail to deliver and are not held accountable	Agbenorku (2012); World Bank (2015); Vian and Norberg (2008); Akokuwebe and Adekanbi (2017); and Vian (2008)	High cost, inappropriate or duplicative drugs and equipment; inappropriate equipment located without consideration of true need; sub-standard equipment and drugs; inequities due to inadequate funds left to provide for all needs
3	Distribution and use of drugs and supplies in service delivery	Theft (for personal use) or diversion (for private sector resale) of drugs/supplies at storage and distribution points; sale of drugs or supplies that were supposed to be free	Amnesty International (2011); Azuh (2012); Vian and Norberg (2008); Stakeholder Democracy Network (2013); Maduke (2013); and Kankeu and Ventelou (2016)	Lower utilization: Patients do not get proper treatment; patients must make informal payments to obtain drugs; interruption of treatment or incomplete treatment, leading to development of anti-microbial resistance
4		Bribes to speed process or gain approval for drug registration, drug quality inspection, or certification of good manufacturing practices; bribes or political considerations influence results of inspections or suppress findings; biased application of sanitary regulations for restaurants, food production and cosmetics; biased application of accreditation, certification or licensing procedures and standards.	Bloom *et al.* (2011); Agyepong (2008); Garuba *et al.* (2009); and Kamorudeen and Bidemi (2012)	Sub-therapeutic or fake drugs allowed on market; marginal suppliers are allowed to continue participating in bids, getting government work; increased incidence of food poisoning; spread of infectious and communicable diseases; poor quality facilities continue to function Incompetent or fake professionals continue
5	Education of health professionals	Bribes to gain place in medical school or other pre-service training; bribes to obtain passing grades; political influence, nepotism in selection of candidates for training opportunities.	Agbenorku (2012); Maduke (2013); Mackey *et al.* (2013); and Maduke (2013)	Incompetent professionals practicing medicine or working in health professions; loss of faith and freedom due to unfair system
6	Medical research	Pseudo-trials funded by drug companies that are really for marketing; misunderstanding of informed consent and other issues of adequate standards in developing countries (including Nigeria).	Garuba *et al.* (2009) and Kamorudeen and Bidemi (2012)	Violation of individual rights; biases and inequities in research
7	Provision of services by medical personnel and other health workers	Use of public facilities and equipment to see private patients; unnecessary referrals to private practice or privately owned ancillary services; absenteeism Informal payments required from patients for services Theft of user fee revenue, other diversion of budget allocations	Turay (2016); Chimezie (2015); Vian and Norberg (2008); Maduke (2013); Saka *et al.* (2016); and Kankeu and Ventelou (2016).	Government loses value of investments without adequate compensation; employees are not available to serve patients, leading to lower volume of services and unmet needs, and higher unit costs for health services actually delivered; reduced utilization of services by patients who cannot pay; impoverishment as citizens use income and sell assets to pay for health care; reduced quality of care from loss of revenue; loss of citizen faith in government

#### Informal payments (bribery)

The terms bribery and informal payments are often used interchangeably in the papers included in the review, and research elsewhere has shown how the various manifestations are often difficult to differentiate, given the challenges of ascertaining the motivation driving the transaction (Gaal and McKee, 2005). However, ‘bribery’ is more often used to refer to offering money or gifts to hasten services or obtain a service while ‘informal payments’ more often refer to paying fees for supposedly free services or paying in kind for health services (Azuh, 2012; Saka *et al.*, 2016).

There were many reports of informal payments or bribes being paid by service users to health workers in cash and/or kind (Garuba *et al.*, 2009; Stakeholder Democracy Network, 2013; Saka *et al.*, 2016; Turay, 2016). They appeared to be more common in remote areas, disproportionately affecting the poor and other vulnerable groups. Bribes were often paid to jump a queue or receive preferential and better quality care, whereas health workers sometimes demanded fees for supposedly free health services (Stakeholder Democracy Network, 2013; Kankeu and Ventelou, 2016). de Sardan (2013) rationalized high prevalence of informal payments in remote areas as due to poverty and low education of many rural dwellers, who may have low expectations of services and have little or no power or opportunity to speak out.

The use of bribes to expedite treatment was often considered as normal. Thus, Saka *et al.* (2016) argued that informal payments and bribery that help patients to avoid bureaucratic bottlenecks rapidly become normalized by patients. They also argued that, once normalized, patients offer unsolicited bribes. Other cases where bribes were reported include doctors refusing to see patients except when a bribe is paid; nurses not monitoring babies until they receive inappropriate payments from their mothers; and staff imposing charges for supposedly free items and interventions (Chimezie, 2015; Turay, 2016).

While it is often argued that poor pay explains demands for bribes by health workers, normalization of bribery by patients themselves calls for explanation. We consider two major antecedents to normalization of bribery in poor regions of Nigeria. First, most sectors in these regions, including policing, power, education and sports, are characterized by arbitrary bribery (Enakhimion, 2011). Accounts are widespread in the media on normalization of the phenomenon in the health sector and throughout society (Enakhimion, 2011; Transparency International, 2017). Second, absence of consumer protection laws removes a potential constraint on health workers acting inconsiderately to patients (Hoffman and Patel, 2017). This lack of protection leaves health service users at the mercy of healthcare providers. Third, many people are uninsured and payment systems are not automated (Aregbesola, 2016), so transactions involve cash that can more easily be diverted than if electronic systems were used.

#### Absenteeism

Absenteeism was a frequently cited form of corruption, especially when health workers are absent to serve personal interests, that going contrary to official norms prescribed for employee behaviour (Vian, 2008; UNDP, 2011; Kamorudeen and Bidemi, 2012; de Sardan, 2013; Maduke, 2013; Chimezie, 2015; Mackey *et al.*, 2016, etc.). It manifests in different forms, including health workers failing to turn up for work, turning up late, being at work but not working and leaving the workplace before closing hours (Diestel *et al.*, 2014). The term ‘absenteeism’ also covers health workers employed in public facilities attending to patients in their private facilities during official hours, or where these public employed health workers engage in contract and consultancy jobs at the expense of their primary employment. This has created situations in which some health workers perform tasks outside the scope of their licenses or expertise (Chimezie, 2015).

There are several factors at work, including weak systems of oversight and sanctions, and protection of some individuals with political or other connections. It is also necessary to consider the association with problems that limit the ability of staff to work effectively in public facilities, including a lack of basic infrastructure and poor transport links (UNDP, 2011; Stakeholder Democracy Network, 2013; Chimezie, 2015).

Measures suggested to curb absenteeism include strict sanctions, rewards linked to being present and improved wages, thereby reducing the perceived need for additional income (Vian and Norberg, 2008). However, there is little evidence of holistic interventions against absenteeism of health workers in AWA countries addressing the spectrum of drivers highlighted above.

#### Theft/diversion of drugs, medical supplies and other public resources

In many situations, it is relatively easy for health workers to divert medical supplies to private facilities or for sale for private gain (Vian and Norberg, 2008; UNDP, 2011; Maduke, 2013; Mackey *et al.*, 2016; Saka *et al.*, 2016; Akokuwebe and Adekanbi, 2017). Some reports described health workers retaining high-quality hospital supplies for their own use whereas selling sub-standard products to clients, or withholding ‘free’ hospital supplies from clients but selling them from their own supplies. Sometimes doctors use public facilities to provide services to their private clients, to the detriment of their public ones (Akinbajo, 2012; Chimezie, 2015). The absence or weak enforcement of consumer protection laws in most AWA countries enables this type of corruption to thrive, as no alarm is raised, and no questions are asked (de Sardan, 2013).

#### Sub-optimal procurement of drugs and other medical equipment

Procurement of medical consumables and equipment is especially susceptible to corruption (Amnesty International, 2011; UNDP, 2011; Kamorudeen and Bidemi, 2012; Maduke, 2013). However, unlike many of the other forms of corruption that involve individual interactions between health care staff and patients in private, this often involves a conspiracy among multiple actors. Thus, storekeepers identify items to be procured, hospital managers approve the need for such supplies and high-ranking officials in ministries or health authorities approve the release or disbursement of funds. These processes are frequently facilitated by pharmaceutical vendors who provide incentives to health administrators and managers to secure procurement contracts, and kickbacks to health workers, motivating them to prescribing and dispensing their branded pharmaceuticals (Vian, 2008). There were also reports of managers bribing officials in health ministries, who send them medical consumables and equipment for sale in their facilities, with the cost of the bribes then reclaimed by charging patients for some of the services that should be free. The underlying reason often relates to the absence of consumer protection mechanisms.

#### Diversion of patients

Doctors may divert patients from public to private health facilities even when public facilities can deliver treatment (Vian and Norberg, 2008). They then charge them for private services. The patients are convinced by the doctors that this improves quality of care (Maduke, 2013). In other cases, they divert their private patients to public facilities where they are treated using government owned equipment and materials, but charged as private patients (Vian and Norberg, 2008; Saka *et al.*, 2016). It is often assumed that this reflects doctors’ poor pay but less frequently often discussed reasons include a high patient–doctor ratio and the relative immunity of doctors from sanctions. Given the shortage of doctors in these countries, they are often seen as doing the public system a favour simply by coming to work at all. Doctors play leading roles in health systems in this region, often in a revolving door linking periods in clinical practice with official roles, providing a disincentive to take definitive action.

### Other types/forms of corruption

Another example of corruption, less frequently reported, relates to employment practices. These include paying bribes to be employed, recruiting staff based on relationships with politically connected persons or relatives/kinsmen; employing ‘ghost workers’ to obtain their wages, renting public facilities for private gain, distributing counterfeit medical consumables, nepotism/favouritism (Maduke, 2013), document forgery, corruption associated with payments for staff promotion, training and deployment; and underpayment of medical staff while diverting resources. Mooketsane and Phirinyane (2017) pointed to inadequate governance, mismanagement and misappropriation of funds as both drivers and manifestations of corrupt practices in the health sector. Agbenorku (2012) described fraud and forgery related to the auditing processes in the Ghanaian National Health Insurance Scheme, with damaging consequences for patients.

### Effects of corruption

Gaitonde *et al.* (2016) summarized the effects of corruption at different levels including general effects, effects on the healthcare system and effects on health outcomes. Health sector corruption has been linked to several adverse health outcomes. Infant and child mortality are estimated to be almost twice as high in countries with high corruption indices than in countries where they are low. Others include inadequate immunization rates, delays in treatments, failure to treat patients, reduced use of public health clinics, reduced satisfaction with care and increased waiting times (Vian and Norberg, 2008; Akpomuvie, 2010; Adegboyega and Abdulkareem, 2012; Agbenorku, 2012; Onotai and Nwankwo, 2012; World Bank, 2015; Turay, 2016). In addition, corruption in the health sector has been blamed for increases in the cost of services, poor staff development, shortages of medical consumables and lack of availability and quality of health service equipment (Akinbajo, 2012; Azuh, 2012). However, although many of these associations are intuitive, few have been subjected to detailed analysis.

Research on how the public view corruption points to a phenomenon seen as being created and perpetuated by health workers, patients and government agents (Azuh, 2012; Maduke, 2013; Stakeholder Democracy Network, 2013; Hoffman and Patel, 2017). Vian (2008) mentioned patients normalizing corrupt practices. However, such normalization did not dispel the negative feelings patients expressed towards the corrupt act as they apportion blames to health workers (Stakeholder Democracy Network, 2013; Hoffman and Patel, 2017). For instance, patients typically blamed doctors for absenteeism and the hospital management for the generally poor care they receive when they visit the facility (Stakeholder Democracy Network, 2013). Thus, while some of the root causes and facilitators of corruption lie in how the government fails health workers, patients are much more liable to hold front-line health workers and their managers accountable.

### Measures to reduce corruption in the health sector

Some of the previously mentioned drivers of corruption in the health sector, such as poor pay of health workers, poor working conditions, a lack of electronic systems for handling money, absence of consumer protection, poor health infrastructure, weak application of rules and deviant social norms should feature as pointers to remedies. However, most recommendations rely on theory rather than empirical evidence. For example, Tormusa and Idom (2016) advocate whistleblowing mechanisms to facilitate reporting of misconduct and corrupt practices. Others advocate establishing fraud control units, internal audits, surveillance systems, attitudinal training, transparent detection and prosecution measures, institutionalizing formal Public–Private Partnership (PPP) models, improved incentives, public sensitization, and Information and Communication Technologies (ICT) for monitoring procurement (Vian, 2008; Maduke, 2013; Turay, 2016; Hanna et al., 2011).

Clearly, the responses should be tailored to the type of corruption. Thus, Mackey *et al.* (2016) advocate community monitoring, enforcement of ethical principles and performance bonuses as ways to curb absenteeism. Making patients aware of official prices of services and consumables, improving work incentives for healthcare workers and de-emphasizing the belief that giving bribes is a norm in healthcare facilities have been suggested as viable means of curtailing informal payments and bribery (Onwujekwe *et al.*, 2010; Maduke, 2013; Agbenorku, 2016; Tormusa and Idom, 2016; Hoffman and Patel, 2017; Mitchell, 2017). Ojiaku (2014) suggested enhanced control mechanisms as a way to tackle fraudulent procurement. Electronic money transfer has been proposed as a way of reducing diversion of co-payments (Vian, 2008; UNDP, 2011; The Association of Chartered Certified Accountants, 2013; Gaitonde *et al.*, 2016; Holeman *et al.*, 2016).

Interventions proposed to reduce theft include attitudinal training for health workers, regular monitoring of stores, store records, and management procedures, improved employment practices, and better work incentives and remuneration of health workers (Garuba *et al.*, 2009; Uzochukwu *et al.*, 2011; Akokuwebe and Adekanbi, 2017; Azuh, 2012; Stakeholder Democracy Network, 2013; Maduke, 2013; Chimezie, 2015). Electronic procurement systems have been proposed to increase transparency (Onwujekwe *et al.*, 2010; UNDP, 2011; Maduke, 2013; The Association of Chartered Certified Accountants, 2013). Adequate and appropriate staffing, surveillance of stores, use of security services and other agencies (multi-stakeholder partnership), timely and frequent reviews of financial records, have been proposed as promoting corruption-free processes more generally (Human Right Watch, 2007; Vian and Norberg, 2008; Onwujekwe *et al.*, 2010; Bloom *et al.*, 2011; UNDP, 2011; Maduke, 2013; The Association of Chartered Certified Accountants, 2013; Holeman *et al.*, 2016).

Unfortunately, few of these proposals are supported by actual evidence of effectiveness. An exception is an ICT-based intervention to monitor payments for health services in Ghana, reducing fraudulent practices (Vian and Norberg, 2008; The Association of Chartered Certified Accountants, 2013). The Ghanaian example may not, however, be easily transferable to other settings. Ghana stands out in Africa for its investment in information technology. The World ICT Development Index rates Ghana in 116th position, with Nigeria and Gambia at 143 and 144 positions, respectively, while Liberia and Sierra Leone were not listed (ITU Data, 2017). Ghana is seventh in Africa’s ICT development index, with none of the other AWA countries in the top 10 (IT News Africa, 2017). Another evidence-based approach to combatting corruption is public sensitization, which raised awareness among Sierra Leonean patients that they had a right to refuse to give bribes (Pay No Bribe n.d.; Anti-Corruption Commission, 2014). These measures offer promise because of their scope for local implementation without the need to obtain consent from higher authorities in the hierarchy or to navigate complex processes.

## Discussion

The growth in the literature on corruption in AWA since 2010 is an indication of its increasing relevance to the region and globally. Once taken for granted, corruption, its causes, and ways to address it, are now attracting considerable attention from researchers and policymakers. This likely reflects growing recognition of the role that corruption plays in impeding progress towards development targets, such as the Millennium Development Goals and now the Sustainable Development Goals, coupled with greater visibility, brought about in part by the publication of corruption perception indices. It is especially high on the agenda in this sub-region now, given poor performance on transparency indices that has focused attention on how weak systems of governance impede health system strengthening.

This review seeks to narrow the gap between evidence on the scale and nature of corruption in this region and action. The adopted conceptualizations (Gaal and McKee, 2004; Vian and Norberg, 2008; Vian, 2008; de Sardan, 2013; Gaitonde *et al.*, 2016) offered basis for structuring and interpreting our findings with key explanations on what factors sustain different forms of corruption in the health sector, and what approaches may offer a promise in beginning to address them. However, the review has a number of limitations. Most obviously, it is dependent on what has been written about a topic that is almost always hidden and, in many cases, actively concealed from public view. As it threatens vested interests, some with substantial power locally and nationally, it is especially difficult to research. In fact, much of what is known about corruption generally, and not only in the health sector, has come from investigative journalism rather than academic research. Although beyond the scope of this review, this raises questions for the health systems research community, including methods that can be used and the challenge of reconciling them with conventional principles of research ethics, based on informed consent by the subjects of the research. This is obviously problematic when the goal of the research is to expose unethical and, in many cases, criminal activity. Added to this is the potential threat to the safety of researchers.

This review is only a first step in addressing a complex area, which our adopted frameworks (Vian and Norberg, 2008; Vian, 2008; Gaitonde *et al.*, 2016) describe as involving powerful actors, social norms, social ties and sensitive areas of health system, that seem difficult to study and tackle. Nevertheless, we begin by providing an understanding of what corruption is in the AWA context. We were able to create a typology of corruption in the health sector and, even though many types of corruption coincide, it seems likely that there will be a need to combine generic measures related to functioning of institutions and adequacy of funding with measures that are specific to the different types of corruption that we identify. Examples include electronic systems to tackle absenteeism, as well as theft and procurement fraud, and public information and awareness raising programmes to challenge normative assumptions about bribery. The issue of public enlightenment is in line with the idea for addressing corruption by giving voice to the weak (Gaal and McKee, 2004) and beginning to challenge informal behaviours that contravene ethical conduct in the health sector (de Sardan, 2013).

Further, it was not possible to derive a quantitative measure of the scale of corruption in the health sector in AWA but it was clear that it is widespread and takes many forms (Vian, 2008). We were able to identify different types and forms of corrupt practices; their drivers, and the consequences of corruption for the lives of service users in the region. The common types of corruption identified from the reviewed studies were bribery, informal payments, absenteeism, theft/diversion of medical supplies and patients, and procurement frauds. However, other corrupt practices, such as document forgery, employment irregularities, corruption in staff training, staff deployment, politically motivated distribution of health facilities and underpayment of medical staff, were also identified.

While different types of corruption identified have a common objective, which is the acquisition of private gain at public expense, the factors that predispose to them vary greatly. These predisposing factors involve a wide variety of people, including health workers, health managers and service users, operating within a system that frequently creates incentives for corrupt behaviour (Hoffman and Patel, 2017). This was based on the work of Gaitonde *et al.* (2016) who believed that poor health system governance is a conduit for corruption to happen unchecked, as well as the Global Corruption Report (2006) and Vian (2008) who argued that collective understanding and inactions among stakeholders in the health sector sustain corrupt practices. Thus, the normative perception of bribery and informal payments by health consumers, dual practice, poor pay and welfare for health staff, service provider–service user power differentials, cash payments, low public awareness levels, scanty monitoring and evaluation processes, and an absence of a procurement procedures, were all identified in reviewed studies as key drivers of corruption in the region. Underlying them, however, is weak health system governance, often identified as the root cause of the endemic nature of corruption in AWA (Gaitonde *et al.*, 2016), with corruption often considered the norm by government agents, clients and health workers, leaving very little room for change (Vian and Norberg, 2008; Vian, 2008). However, as argued by Khan and corroborating the findings of this review, there may be also a need to depart from the normative government-driven anti-corruption measures, and shift the focus to addressing the behaviours and interests of front-line health workers and facility managers, while placing them in the context of local political structures (Khan, 2017). The deep-seated problems of weak governance combined with poor incentives for health systems actors to change the status-quo means that there is little evidence to support particular anti-corruption interventions, consistent with the systematic review by Gaitonde *et al.* (2016).

By systematically describing the nature of corruption in health sectors in AWA, we hope to raise it on the national and global political agenda and encourage governments and non-governmental organizations—in the region and beyond—to develop interventions that can tackle the different types of corruption that we have described.

*Ethical approval.* It is a systematic review, no human subject was used.
